# Omega-3 Fatty Acids Interact with *DPP10* Region Genotype in Association with Childhood Atopy

**DOI:** 10.3390/nu15102416

**Published:** 2023-05-22

**Authors:** Kathleen A. Lee-Sarwar, Kasper Fischer-Rasmussen, Klaus Bønnelykke, Hans Bisgaard, Bo Chawes, Rachel S. Kelly, Jessica Lasky-Su, Robert S. Zeiger, George T. O’Connor, Leonard B. Bacharier, Vincent J. Carey, Nancy Laranjo, Augusto A. Litonjua, Scott T. Weiss

**Affiliations:** 1 Channing Division of Network Medicine, Brigham and Women’s Hospital and Harvard Medical School, Boston, MA 02115, USA; 2 Division of Allergy and Clinical Immunology, Brigham and Women’s Hospital and Harvard Medical School, Boston, MA 02115, USA; 3 Copenhagen Prospective Studies on Asthma in Childhood (COPSAC), Herlev and Gentofte Hospital, University of Copenhagen, 2820 Gentofte, Denmark; 4 Department of Clinical Science, Kaiser Permanente Bernard J. Tyson School of Medicine, Pasadena, CA 91101, USA; 5 Pulmonary Center and Department of Medicine, Boston University School of Medicine, Boston, MA 02118, USA; 6 Division of Pediatric Allergy, Immunology and Pulmonary Medicine, Department of Pediatrics, Monroe Carell Jr. Children’s Hospital at Vanderbilt, Vanderbilt University Medical Center, Nashville, TN 37212, USA; 7 Division of Pediatric Pulmonary Medicine, Golisano Children’s Hospital at Strong, University of Rochester Medical Center, Rochester, NY 14612, USA

**Keywords:** omega-3, polyunsaturated fatty acids, asthma, allergy, genotype

## Abstract

Associations of omega-3 fatty acids (*n*-3) with allergic diseases are inconsistent, perhaps in part due to genetic variation. We sought to identify and validate genetic variants that modify associations of *n*-3 with childhood asthma or atopy in participants in the Vitamin D Antenatal Asthma Reduction Trial (VDAART) and the Copenhagen Prospective Studies on Asthma in Childhood 2010 (COPSAC). Dietary *n*-3 was derived from food frequency questionnaires and plasma *n*-3 was measured via untargeted mass spectrometry in early childhood and children aged 6 years old. Interactions of genotype with *n*-3 in association with asthma or atopy at age 6 years were sought for six candidate genes/gene regions and genome-wide. Two SNPs in the region of *DPP10* (rs958457 and rs1516311) interacted with plasma *n*-3 at age 3 years in VDAART (*p* = 0.007 and 0.003, respectively) and with plasma *n*-3 at age 18 months in COPSAC (*p* = 0.01 and 0.02, respectively) in associationwith atopy. Another *DPP10* region SNP, rs1367180, interacted with dietary *n*-3 at age 6 years in VDAART (*p* = 0.009) and with plasma *n*-3 at age 6 years in COPSAC (*p* = 0.004) in association with atopy. No replicated interactions were identified for asthma. The effect of *n*-3 on reducing childhood allergic disease may differ by individual factors, including genetic variation in the *DPP10* region.

## 1. Introduction

Increases in the prevalence of allergic diseases, including aeroallergen sensitization and asthma, have co-occurred over recent decades with dietary changes in industrialized countries. These changes have included reductions in omega-3 polyunsaturated fatty acid (*n*-3 PUFA) intake [1]. *N*-3 PUFA has well-described anti-inflammatory and pro-resolving immunologic effects [2]. These findings suggest a potential causal role of PUFA in allergic disease pathobiology. However, reported associations of PUFA with atopy and asthma in childhood have been inconsistent [3,4].

Although the diet is the primary source of PUFA, bioactive metabolites can be produced endogenously from α-linolenic acid and linoleic acid via several desaturation and elongation steps. Human genetic variants in this pathway have been previously linked to both PUFA levels and to differential associations between PUFA and several non-allergic diseases, including cholesterol and triglyceride levels, cancer, and heart disease [5]. When it comes to allergic diseases, investigations of the role of genetic variation in PUFA metabolism have focused almost entirely on the genes *FADS1* and *FADS2*, which encode rate-limiting steps in both *n*-3 and omega-6 (*n*-6) PUFA metabolic pathways [6,7,8,9,10,11,12]. Emerging evidence also implicates *ELOVL6*, which encodes another enzyme involved in PUFA metabolism, in asthma pathophysiology [13].

In this ancillary observational study of data from the Vitamin D Antenatal Asthma Reduction Trial (VDAART), we tested the hypothesis that the effects of *n*-3 PUFA on the risk of childhood atopy and asthma are modified by host genotypes. We investigated target genes including, but not limited to, the *FADS* region. We sought replication of the key findings in the Copenhagen Prospective Studies on Asthma in Childhood 2010 (COPSAC) birth cohort.

## 2. Materials and Methods

Study Participants and Setting: Detailed methods are provided in the Supplementary Materials. Analyses were performed using data from the VDAART clinical trial (https://clinicaltrials.gov/ct2/show/NCT00920621, accessed on 4 May 2023) and replication of the findings was sought in data from the COPSAC clinical trial. VDAART [14] is a double-blind placebo-controlled multi-site study on the effects of prenatal vitamin D supplementation on asthma and allergy in offspring. The study protocol was approved by the institutional review boards at each participating institution and at Brigham and Women’s Hospital. COPSAC [6] is a Danish birth cohort study on the development of asthma and allergies. The COPSAC2010 study was approved by the local ethics committee with a separate approval for the vitamin D administration during pregnancy RCT, by the Danish Data Protection Agency, and by the Danish Health and Medicines Authority. All participants provided written informed consent.

Dietary *n*-3 PUFA (VDAART): Food frequency questionnaire (FFQ) responses were used to estimate daily calorie and *n*-3 PUFA intake (eicosapentaenoic acid (EPA) + docosahexaenoic acid (DHA)) when the offspring were 3 and 6 years old [15,16]. To account for total energy intake, nutrient density was calculated for each nutrient by dividing the nutrient intake by the total calorie intake and all analyses of nutrient densities included estimated calorie intake as a covariate [17]. *n*-3 nutrient density was analyzed as a continuous variable.

Plasma *n*-3 PUFA (VDAART and COPSAC): Participants in VDAART and COPSAC provided plasma samples for metabolomics analysis at age 3 and 6 years (VDAART) and 18 months and 6 years (COPSAC). Metabolomic profiling by mass spectrometry was performed at Metabolon, Inc. (Research Triangle Park, Morrisville, NC, USA). A summary variable was created for *n*-3 plasma PUFA by taking the sum of EPA and DHA. Plasma *n*-3 was analyzed as a continuous variable after log-normalization and standardization.

Clinical Outcomes and Covariates: Asthma and atopy were ascertained at age 6 years as described in the Supplementary Materials, with atopy criteria based on aeroallergen-specific IgE measurements in both cohorts and skin testing data additionally available for the COPSAC participants.

Genotyping and Candidate Gene Selection: Genotyping was performed in VDAART and COPSAC participants using the Illumina Infinium HumanOmniExpressExome Bead chip. We selected the candidate genes/gene regions *FADS* region, *ELOVL2*, *ELOVL5*, *DPP10*, *PTGES*, and *PTGS2* based on evidence of associations with both PUFA and with asthma and allergy, with details available in the Supplementary Materials.

Statistical Analysis: Model covariates were selected a priori. Covariates in the analyses of the VDAART data were sex, race (black vs. non-black), study center, VDAART treatment assignment and the top four genotype PCs. Covariates in COPSAC data were sex, COPSAC treatment assignment and the top four genotype PCs. Analyses of dietary *n*-3 PUFA were additionally adjusted for estimated daily calorie intake. We performed logistic regression analyses under an additive genetic model including the main and interaction effects of genotype and *n*-3 PUFA. These analyses were performed on SNPs within 50,000 base pairs of candidate genes/gene regions in the targeted analysis, and on a genome-wide basis. Findings were considered statistically significant on a genome-wide basis if they reached a level suggestive of statistical significance (*p* < 1 × 10^−5^) in VDAART and reached a level of statistical significance with *p* < 0.01 and the same direction of association in COPSAC. The candidate gene analysis results were considered statistically significant if they yielded *p* < 0.01 in VDAART and *p* < 0.05 in COPSAC with the same direction of association. We also applied a more stringent false discovery rate (FDR) correction for multiple testing. Removal of statistically significant SNPs within 1000 kB regions that were in high LD (r^2^ > 0.05) was performed by LD clumping.

## 3. Results

### 3.1. Plasma and Dietary n-3 PUFA

**Figure 1:**
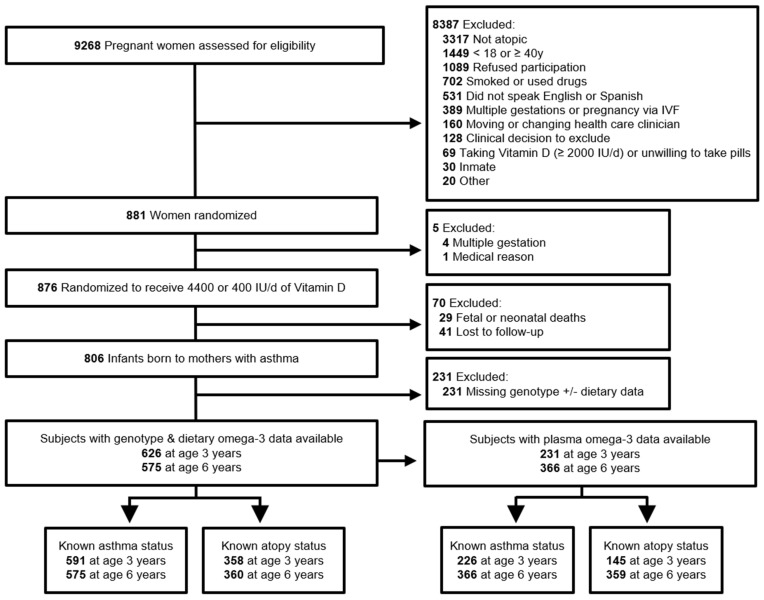
Flow diagram of VDAART subjects included in study.

A total of 626 and 575 VDAART participants had available genotype and dietary *n*-3 measurements at ages 3 and 6 years, respectively (Figure 1). Of these, 231 (37% of 626) also had plasma *n*-3 measurements at age 3 years and 366 (64% of 575) at age 6 years. Dietary *n*-3 levels were similar between subjects with and without available plasma *n*-3 (*t*-test *p* = 0.24 at age 3 years and 0.94 at age 6 years). Plasma *n*-3 and dietary *n*-3 measurements exhibited small but statistically significantly correlations at both ages 3 and 6 years (Pearson *rho* = 0.14, *p* = 0.04 at age 3 years; *rho* = 0.21, *p* < 0.001 at age 6 years; linear regression adjusted for daily calorie intake *p* = 0.02 at age 3 years and *p* < 0.01 at age 6 years, Figure S1), similar to previously reported small-to-moderate correlations between circulating and dietary PUFA [18]. Accordingly, we analyzed plasma and dietary *n*-3 separately in subsequent analyses.

Of the 87 food frequency questionnaire items, several food items exhibited correlations with plasma and/or dietary *n*-3 measurements, including eggs (Spearman rho range 0.06–0.20), non-fried fish (Spearman rho range 0.19–0.26) and fried fish (Spearman rho range = 0.05–0.17, Figure S2).

### 3.2. Subject Characteristics

Baseline characteristics of the VDAART study subjects are displayed in Table 1 and Table 2 and were balanced between subjects with plasma or dietary *n*-3 values above vs. below the median, although dietary *n*-3 at age 3 years was more likely to be above the median in black than white subjects (Table 1). There was limited overlap between asthma and atopy phenotypes; out of 199 subjects with atopy, 42 (21%) also had asthma.

We sought replication of the VDAART results using data from 583 participants in the COPSAC study with available genotype data and plasma *n*-3 measurements at either age of 18 months or 6 years. The frequency of atopy and asthma outcomes in VDAART and COPSAC participants are provided in Table 2. Dietary *n*-3 measurements were not available in COPSAC. Of note, all COPSAC participants were white, while VDAART participants were racially diverse (21% white, 40% black, 39% other race, Table 3). We stratified findings in VDAART by black vs. non-black race both as a sensitivity analysis of significant results and to seek associations that may be missed in the whole-sample analysis due to genetic heterogeneity. Dichotomous stratification by black vs. non-black race was selected to preserve sample size and because, based on the distribution of the top two genotype principal components, this variable better reflected genetic variation than white vs. non-white race (Figure S3).

### 3.3. Associations of n-3 PUFA with Asthma and Atopy

Plasma *n*-3 at age 6 years was inversely associated with asthma in the analyses adjusted for sex, race, study center and VDAART treatment assignment (adjusted logistic regression OR = 0.45, 95% CI 0.23–0.82, *p* = 0.01), but was not associated with atopy (OR 0.85, 95% CI 0.54–1.32, *p* = 0.48). Dietary *n*-3 at age 6 years exhibited inverse but non-significant associations with both asthma (OR = 0.97, 95% CI 0.93–1.01, *p* = 0.15) and atopy (OR = 0.98, 95% CI 0.95–1.01, *p* = 0.21) in models additionally adjusted for total daily calorie intake. At age 3 years, neither plasma nor dietary *n*-3 were associated with asthma or atopy (all *p* > 0.05). In COPSAC participants, plasma *n*-3 at age 18 months or 6 years was not associated with either asthma or atopy (all *p* > 0.05 in logistic regression models adjusted for sex and treatment assignment).

### 3.4. Targeted Analysis

In the SNP x *n*-3 interaction analyses limited to six targeted genes selected based on the literature review (*FADS*, *ELOVL2*, *ELOVL5*, *DPP10*, *PTGES*, and *PTGS2*), out of 6025 SNPs that entered into the analysis, a total of 174 SNPs passed our predetermined significance threshold (*p* < 0.01) in VDAART for the outcome of asthma (61 SNPs interacted with dietary *n*-3 at age 3 years; 9 with dietary *n*-3 at age 6 years; 40 with plasma *n*-3 at age 3 years; 64 with dietary *n*-3 at age 6 years), and 92 SNPs were significant for the outcome of atopy (21 SNPs interacted with dietary *n*-3 at age 3 years; 28 with dietary *n*-3 at age 6 years; 35 with plasma *n*-3 at age 3 years; 8 with dietary *n*-3 at age 6 years). Of the SNPs passing significance thresholds in VDAART (*p* < 0.01), 21 also passed the significance threshold in COPSAC (*p* < 0.05; 9 and 11 SNPs interacted with *n*-3 at age 18 months in association with atopy as defined by aeroallergen skin testing, respectively, and 1 SNP interacted with *n*-3 at age 6 years in association with atopy by skin testing). To reduce multiple testing of highly correlated SNPs, LD clumping was performed using an r^2^ threshold of 0.05 such that pairs of SNPs within a 1000 kB window with r^2^ > 0.05 were clumped together and the most statistically significant among them was carried forward while the others were excluded. This identified a total of seven independent SNPs that passed the *p* value significance thresholds in both cohorts, including six in the region of the gene *DPP10* and one in the region of the gene *ELOVL2*. Of these seven SNPs, three demonstrated concordant directions of association in both VDAART and COPSAC participants in covariate-adjusted logistic regression models of *n*-3 associations with clinical outcomes, stratified by genotype (Table 4). All three replicated SNPs were in the region of the gene *DPP10*, and all exhibited associations with the outcome of atopy as defined by IgE testing in VDAART and skin testing in COPSAC, with no significant interactions found with the outcome of asthma. While all three replicated SNPs met our prespecified criteria for statistical significance, they were no longer statistically significant after more stringent correction for multiple testing (FDR > 0.05).

**Figure 2:**
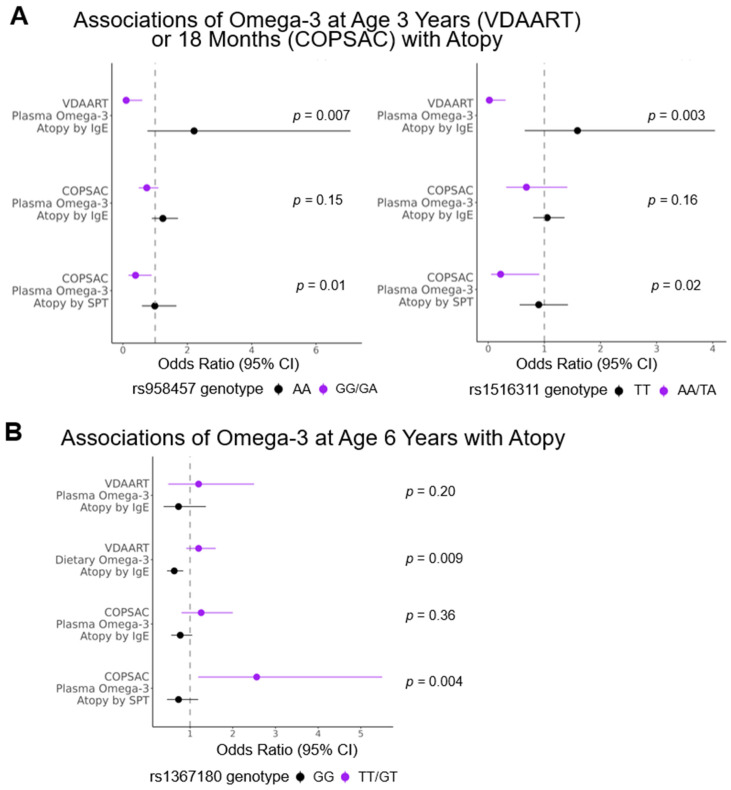
Associations of *n*-3 and atopy in VDAART and COPSAC participants stratified by rs958457 and rs1516311 genotypes (**A**) and rs1367180 genotype (**B**). Odds ratios, 95% confidence intervals and *p* values for interaction tests from covariate-adjusted logistic regression analyses under an additive genetic model are provided. Abbreviations: SPT = skin prick test.

SNPs rs958457 and rs1516311, both *DPP10* intron variants, exhibited interactions with plasma *n*-3 at age 3 years in VDAART (*p* = 0.007 and 0.003, respectively) and with plasma *n*-3 at age 18 months in COPSAC (*p* = 0.01 and 0.02, respectively) associated with the outcome of atopy. Specifically, individuals with at least one minor allele (G for rs958457, *n* = 61 in VDAART and *n* = 188 in COPSAC; A for rs1516311, *n* = 31 in VDAART and *n* = 92 in COPSAC) exhibited a negative association of *n*-3 with atopy and individuals homozygous for the major allele (rs958457: *n* = 84 in VDAART and *n* = 243 in COPSAC; rs1516311: *n* = 114 in VDAART and *n* = 339 in COPSAC) exhibited a positive association of *n*-3 with atopy (Table 4, Figure 2). These two SNPs demonstrated a small positive correlation with one another (allele dosage Pearson rho = 0.32, *p* < 0.01).

**Figure 3:**
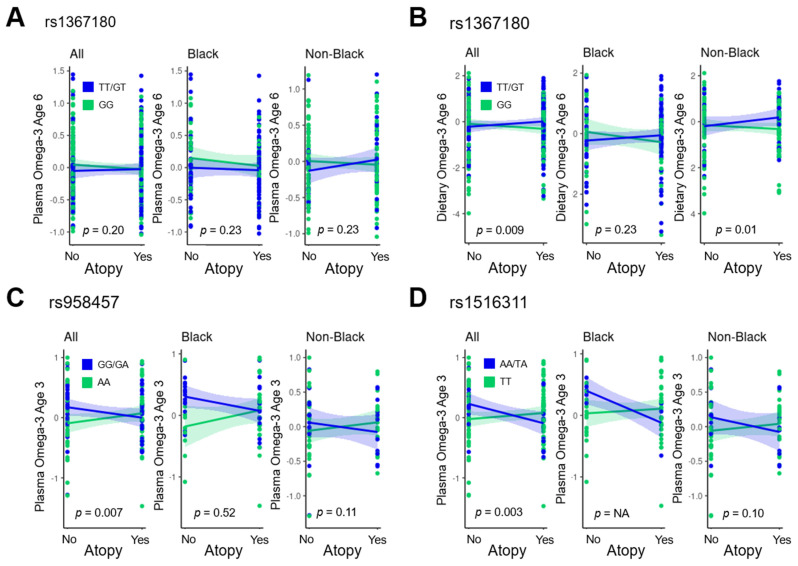
**Associations of *n*-3 with atopy stratified by significant *DPP10* region SNP genotypes and by race in VDAART participants.** (**A**) Associations of plasma omega-3 at age 6 years with atopy, stratified by rs1367180 genotype. (**B**) Associations of dietary omega-3 at age 6 years with atopy, stratified by rs1367180 genotype. (**C**) Associations of plasma omega-3 at age 3 years with atopy, stratified by rs958457 genotype. (**D**) Associations of plasma omega-3 at age 3 years with atopy, stratified by rs1516311 genotype. Interaction *p* values from logistic regression analyses under an additive genetic model are shown. *p* = NA indicates insufficient sample size for analysis.

The third significant SNP, rs1367180, 21,399 bases upstream of *DPP10*, exhibited an interaction with dietary *n*-3 at age 6 years in VDAART (*p* = 0.009) and with plasma *n*-3 at age 6 years in COPSAC (*p* = 0.004). Specifically, individuals with at least one minor allele (T, *n* = 163 in VDAART and *n* = 151 in COPSAC) exhibited a positive association of *n*-3 with atopy and individuals homozygous for the major allele (*n* = 197 in VDAART and *n* = 301 in COPSAC) exhibited a negative association of *n*-3 with atopy (Table 4, Figure 2). This *DPP10* region SNP did not exhibit a significant interaction with plasma *n*-3 for atopy in VDAART (*p* = 0.20 for all subjects, *p* = 0.23 for black subjects, *p* = 0.23 for non-black subjects), although similar directions of association were observed, particularly among non-black subjects (Figure 2 and Figure 3A).

In addition to aeroallergen skin prick testing, COPSAC participants underwent aeroallergen-specific IgE measurement. There were 491 COPSAC participants with data available on atopy by skin testing, 475 with available IgE measurements and 430 with both. There were 34 participants who met the criteria for atopy by skin testing and 89 by IgE measurement. Most (28 of 34, 82%) of those who met the criteria for atopy by skin testing also met the criteria for atopy by IgE measurement. A smaller proportion of COPSAC subjects exhibited atopy as defined by at least one positive skin prick test compared to those with atopy as defined by at least one elevated allergen-specific IgE test (6% vs. 15%, Table 3). Analyses of the association between omega-3 and atopy by allergen-specific IgE in COPSAC participants, stratified by genotypes for the three significant *DPP10* region SNPs (rs958457, rs1516311 and rs136718), yielded similar results to those for the outcome of atopy by skin prick testing (Table 4, Figure 2).

Omega-6 PUFA could mechanistically compete with *n*-3 or confound associations between *n*-3 and atopy. Therefore, we performed sensitivity analyses adjusting for omega-6 PUFA and found that all three significant interactions between *DPP10* region SNPs and *n*-3 on atopy were preserved (all *p* < 0.01).

In the analyses stratified by race in VDAART, associations were largely preserved, although the interaction terms reached a nominal level of statistical significance only in non-black subjects for the interaction of *n*-3 at age 6 years and rs1367180 (*p* = 0.01, *p* range otherwise 0.05–0.11 in race-stratified analyses with sample sizes insufficient to estimate interaction of *n*-3 and rs1516311 in black subjects, Figure 3). However, the analyses stratified by race had a limited sample size and should be regarded as exploratory.

In the main effect adjusted linear and logistic regression models, none of the three replicated *DPP10* region SNPSs were associated with either plasma *n*-3, dietary *n*-3 or atopy (*p* > 0.05 for all analyses in VDAART and COPSAC), with the following exception: rs948457 was inversely associated with atopy based on IgE testing in COPSAC (OR = 0.62, 95% CI 0.40–0.97, *p* = 0.04).

A *DPP10* SNP rs11693320 that was previously reported to interact with *n*-3 PUFA regarding lung function (FVC) [19] exhibited no significant interactions with *n*-3 PUFA for either atopy or asthma (all *p* > 0.05), nor was a *FADS* region SNP (rs1535) that was previously found to interact with dietary *n*-3 intake associated with incident childhood asthma [8] and with prenatal fish oil supplementation associated with offspring asthma [6]. The three significant *DPP10* SNPs identified in our analysis correlated poorly with rs11693320 (allele dosage Pearson correlation range −0.06–0.03, all *p* > 0.05; linkage disequilibrium r^2^ range 0.001–0.006). Likewise, these SNPs exhibited poor correlation in the data from the 1000 Genomes Project (R^2^ range = 0.002–0.004), suggesting that these two SNPs are not in linkage disequilibrium [20].

We found no other significant interactions between *n*-3 and other candidate genes or gene regions including *FADS*, *ELOVL2*, *ELOVL5*, *PTGES* and *PTGS2*. For the asthma outcome, there were no candidate gene SNPs that reached our threshold for significant replication in the VDAART and COPSAC cohorts.

### 3.5. Genome-Wide Analysis

Manhattan plots of genome-wide association study results are shown in Figure S4. In the genome-wide association analyses of interactions with *n*-3, no SNPs reached our threshold for significant replication in the VDAART and COPSAC cohorts for either atopy or asthma outcomes.

## 4. Discussion

Previously reported associations between *n*-3 and allergic outcomes have been inconsistent [3,4]. This may be due in part to genetic variation between study populations, including in the region of *DPP10*. We identified three SNPs in the region of the gene *DPP10* that were associated with differential associations of *n*-3 PUFA with atopy in 6-year-old children. These findings occurred both in a racially diverse United States sample (VDAART) and in a Caucasian sample of Danish children (COPSAC). Specifically, rs1367180, upstream of *DPP10,* modified the association of *n*-3 at age 6 years with atopy, and rs958457 and rs1516311 modified the association of *n*-3 at age 18 months (in COPSAC) and 3 years (in VDAART) with atopy. The latter two SNPs, rs958457 and rs1516311, exhibited a small positive correlation. Therefore, these two SNPs may not represent independently associated SNPs but may instead mark a region of possible casual significance. The finding of different *DPP10* region SNPs modifying *n*-3 associations at different ages suggests the complex regulation of expression in this region, but consistent potential import of *DPP10* across childhood.

The gene *DPP10* has been associated with asthma in prior studies [21,22,23,24]. It encodes dipeptidyl peptidase-like 10, a membrane protein that binds specific voltage-gated potassium channels and modulates potassium trafficking. It has been thought to possibly impact neurologic control of smooth muscle function in asthma [19]. *DPP10* has not been, to our knowledge, previously linked directly to PUFA metabolism. However, a genotype x *n*-3 interaction study of pulmonary function in adults found an interaction for forced vital capacity (FVC) between the *n*-3 docosahexaenoic acid (DHA) and a SNP in DPP10 [19]. This previously identified SNP differed from the SNP identified in our analysis and the two do not appear to be in linkage disequilibrium either in our sample or using the 1000 Genomes data. This suggests that they may have independent or age-dependent effects on the associations of *n*-3 fatty acids with pulmonary function or allergic diseases.

The prior literature on genetic variants as effect modifiers of *n*-3 associations with allergic outcomes have focused predominantly on the *FADS* region. *FADS1* and *FADS2* encode rate-limiting enzymes in the metabolism of both *n*-3 and *n*-6 PUFA and are located in the *FADS* region on chromosome 11. Multiple studies have found associations between *FADS* SNPs and both PUFA levels and eczema [9,10,11]. Prior studies have also found that the *FADS* genotype modifies associations of circulating PUFA or dietary PUFA intake with hay fever [7], eczema [7,12], and asthma [6,7,8]. We were not able to replicate previously reported findings of *FADS* variant interactions with *n*-3 on asthma or atopy, and this may have been a result of our limited power due to the small sample size. Our power was likely further reduced by the presence of genetic heterogeneity in the racially and ethnically diverse VDAART study sample, although this feature is a strength in that it increases the generalizability of our findings.

Our study was subject to additional limitations. Plasma *n*-3 measurements were based on untargeted metabolomics profiling and may be less accurate than targeted, absolute quantification of circulating *n*-3. There were significant differences between the COPSAC and VDAART study populations beyond distributions of race/ethnicity, including differences in the earlier time point of *n*-3 measurement (age 18 months in COPSAC and age 3 years in VDAART). Additionally, VDAART study subjects were at elevated genetic risk of atopy and COPSAC subjects were unselected healthy children. Finally, the COPSAC study included a randomized clinical trial of fish oil supplementation during pregnancy, which could impact *n*-3 homeostasis in offspring, although the results were adjusted for trial treatment assignment. Greater harmonization of study populations and clinical outcomes may have increased our ability to replicate the results. Our sample size was relatively small, and this may have reduced the precision of our results and contributed to the failure of our significant results to survive a more stringent correction for multiple testing. However, given the presence of directionally consistent associations in two cohorts passing our prespecified threshold for statistical significance, we believe our findings are worthy of future study. Finally, fatty acids are oxidized over time and the accuracy of our *n*-3 measurements may have been reduced due to the sample storage time. However, this measurement bias is expected to be non-differential by genotype, *n*-3 level and clinical outcomes.

In conclusion, while limited by the relatively small sample size and associative (not causal) findings, our results add to the existing literature suggesting that *n*-3 PUFA may be effective in reducing childhood allergic disease. This intervention may differ based on individual factors, including genetic variation. While the emphasis in this subject area has been on the *FADS* region, our data suggest that variation in the *DPP10* region may also be of relevance in precision medicine approaches to asthma management through *n*-3 PUFA supplementation.

## Figures and Tables

**Table 1:** **Association of plasma and dietary *n*-3 at age 6 years with baseline characteristics.** Baseline characteristics are also shown for the overall VDAART cohort. *p* values are for the *t*-test for BMI and otherwise for the chi-square test. Percentages may not sum to 100 due to rounding. BMI z scores are based on the Centers for Disease Control and Prevention (CDC) growth charts.

		Dietary Omega-3			Plasma Omega-3			
	Analyzed Subjects (*n* = 575)	≤Median (*n* = 288)	>Median (*n* = 287)	*p* Value	≤Median (*n* = 183)	>Median (*n* = 183)	*p* Value	VDAART Cohort (*n* = 806)
**Sex—number (%)**				0.80			0.40	
** Male**	300 (52)	148 (51)	152 (53)		102 (56)	93 (51)		421 (52)
** Female**	275 (48)	140 (49)	135 (47)		81 (44)	90 (49)		385 (48)
**Race—number (%)**				1.00			0.50	
** Black**	272 (48)	137 (48)	135 (47)		80 (44)	90 (49)		390 (48)
** White**	191 (34)	95 (33)	96 (33)		65 (36)	57 (31)		265 (33)
** Other**	112 (19)	56 (19)	56 (20)		38 (21)	36 (20)		151 (19)
**Hispanic—number (%)**	183 (32)	91 (32)	92 (32)	1.00	69 (38)	64 (35)	0.70	273 (34)
**VDAART treatment group—number (%)**				0.30			0.50	
** 4400 IU/day vitamin D**	294 (51)	154 (53)	140 (49)		87 (48)	95 (52)		405 (50)
** 400 IU/day vitamin D**	281 (49)	134 (47)	147 (51)		96 (52)	88 (48)		401 (50)
**Study Center—number (%)**				0.40			0.90	
** Boston**	137 (24)	65 (23)	72 (25)		48 (26)	49 (27)		240 (30)
** St. Louis**	243 (42)	130 (45)	113 (39)		72 (39)	67 (37)		292 (36)
** San Diego**	195 (34)	93 (32)	102 (36)		63 (34)	67 (37)		274 (34)
**Maternal education—number (%)**				0.50			0.70	
** <High school**	75 (13)	37 (13)	38 (13)		29 (16)	24 (13)		100 (12)
** High school or ** ** technical school**	161 (28)	84 (29)	77 (27)		55 (30)	54 (30)		241 (30)
** Some level of college education**	133 (23)	72 (25)	61 (21)		42 (23)	38 (21)		192 (24)
** College graduate or ** ** higher**	206 (36)	95 (33)	111 (39)		57 (31)	67 (37)		273 (34)
**Birth by cesarean section—number (%)**	166 (29)	89 (31)	77 (27)	0.30	52 (28)	48 (26)	0.70	239 (30)
**Preterm birth < 37 weeks’ gestation—number (%)**	50 (9)	28 (10)	22 (8)	0.50	19 (10)	11 (6)	0.20	71 (9)
**Exclusive breastfeeding for first 4 months of life—number (%)**	185 (34)	87 (32)	98 (37)	0.30	61 (36)	67 (39)	0.60	247 (33)
**BMI (kg/m^2^) at age 6 years—mean (SD)**	17 (2.8)	17 (2.6)	17 (2.9)	0.40	17 (2.6)	17 (2.8)	0.30	17 (2.9)
**BMI z score at age 6 years—mean (SD)**	0.54 (1.16)	0.62 (1.05)	0.45 (1.26)	0.11	0.55 (1.22)	0.45 (1.17)	0.42	0.57 (1.19)

Missing data: breastfeeding missing for 38 subjects; BMI missing for 112 subjects.

**Table 2:** **Association of plasma and dietary *n*-3 at age 3 years with baseline characteristics**. Baseline characteristics are also shown for the overall VDAART cohort. *p* values are for the *t*-test for BMI and otherwise for the chi-square test. Percentages may not sum to 100 due to rounding. BMI z scores are based on the Centers for Disease Control and Prevention (CDC) growth charts.

		Dietary Omega-3			Plasma Omega-3			
	Analyzed Subjects (*n* = 626)	≤Median (*n* = 313)	>Median (*n* = 313)	*p* Value	≤Median (*n* = 116)	>Median (*n* = 115)	*p* Value	VDAART Cohort (*n* = 806)
**Sex—number (%)**				0.63			0.74	
** Male**	325 (52)	166 (53)	159 (51)		60 (52)	63 (55)		421 (52)
** Female**	301 (48)	147 (47)	154 (49)		56 (48)	52 (45)		385 (48)
**Race—number (%)**				**0.03**			0.26	
** Black**	299 (48)	135 (43)	164 (52)		54 (47)	54 (47)		390 (48)
** White**	201 (32)	115 (37)	86 (27)		41 (35)	33 (29)		265 (33)
** Other**	126 (20)	63 (20)	63 (20)		21 (18)	28 (24)		151 (19)
**Hispanic—number (%)**	206 (33)	113 (36)	93 (30)	0.11	38 (33)	47 (41)	0.25	273 (34)
**VDAART treatment group—number (%)**				0.07			0.84	
** 4400 IU/day vitamin D**	312 (50)	168 (54)	144 (46)		58 (50)	55 (48)		405 (50)
** 400 IU/day vitamin D**	214 (50)	145 (46)	169 (54)		58 (50)	60 (52)		401 (50)
**Study Center—number (%)**				0.51			0.18	
** Boston**	157 (25)	79 (25)	78 (25)		22 (19)	24 (21)		240 (30)
** St. Louis**	256 (41)	134 (43)	122 (39)		56 (48)	42 (37)		292 (36)
** San Diego**	213 (34)	100 (32)	113 (36)		38 (33)	49 (43)		274 (34)
**Maternal education—number (%)**				0.61			0.18	
** <High school**	84 (13)	44 (14)	40 (13)		11 (10)	18 (16)		100 (12)
** High school or** ** technical school**	184 (29)	94 (30)	90 (29)		32 (38)	27 (24)		241 (30)
** Some level of college education**	142 (23)	64 (20)	78 (25)		35 (30)	24 (21)		192 (24)
** College graduate or** ** higher**	216 (35)	111 (36)	105 (34)		38 (33)	46 (40)		273 (34)
**Birth by cesarean section—number (%)**	180 (29)	90 (29)	90 (29)	1.00	41 (35)	35 (30)	0.51	239 (30)
**Preterm birth < 37 weeks’ gestation—number (%)**	49 (8)	31 (10)	18 (6)	0.07	11 (9)	6 (5)	0.32	71 (9)
**Exclusive breastfeeding for first 4 months of life—number (%)**	196 (33)	94 (32)	102 (35)	0.50	36 (34)	34 (31)	0.77	247 (33)
**BMI (kg/m^2^) at age 3 years—mean (SD)**	16.6 (1.7)	16.6 (1.8)	16.5 (1.7)	0.52	16.9 (2.1)	16.5 (1.8)	0.12	16.6 (1.8)
**BMI z score at age 3 years—mean (SD)**	0.37 (1.14)	0.39 (1.17)	0.35 (1.12)	0.64	0.58 (1.24)	0.32 (1.17)	0.10	0.40 (1.16)

Missing data: mode of delivery missing for 1 subject; breastfeeding missing for 40 subjects; BMI missing for 63 subjects.

**Table 3:** Characteristics of VDAART and COPSAC participants with genotype and either plasma or dietary *n*-3 data available. Numbers (%) are displayed for each characteristic. Minor allele frequencies (MAF) and Hardy–Weinberg Equilibrium (HWE) *p* values for relevant genotypes are shown, where HWE *p* > 1 × 10^−6^ indicates no deviation from expected Mendelian genetic proportions and is a routine quality control genotyping requirement.

	VDAART [14] (*n* = 575)	COPSAC [6] (*n* = 583)
Male sex	327 (52)	300 (51)
White race	204 (33)	583 (100)
Black race	303 (48)	0 (0)
Other race	121 (19)	0 (0)
Asthma	107 (18)	37 (6)
Atopy	199 (55)	34 (6) by skin testing 89 (15) by serum specific IgE
Offspring atopy risk in recruitment population	Elevated genetic risk	General population
rs958457 MAF	0.23	0.24
rs958457 HWE *p* value	0.91	1.00
rs1516311 MAF	0.08	0.21
rs1516311 HWE *p* value	0.13	0.55
rs1367180 MAF	0.26	0.19
rs1367180 HWE *p* value	0.11	0.60

Missing data: atopy status unknown in 274 VDAART and 102 (skin prick)/110 (IgE) COPSAC subjects. Asthma status unknown in 35 VDAART and 28 COPSAC subjects.

**Table 4:** **SNPs that interacted with *n*-3 regarding allergic outcomes with concordant directions of associations in both VDAART (*p* < 0.01) and COPSAC (*p* < 0.05) in the region of *DPP10*.** Adjusted odds ratios and 95% confidence intervals from genotype-stratified logistic regression models are displayed for the associations of *n*-3 with atopy. Covariates in analyses of VDAART data were sex, race (black vs. non-black), study center, VDAART treatment assignment and the top four genotype PCs. Covariates in COPSAC data were sex, COPSAC treatment assignment and the top four genotype PCs.

						Interaction *p* Value	0 Minor Alleles			1 or 2 Minor Alleles		
CHR	ID	VDAART *n*-3	*n*-3 Time Point	Outcome	Minor Allele		VDAART Results	COPSAC Results (Atopy by IgE)	COPSAC Results (Atopy by SPT)	VDAART Results	COPSAC Results (Atopy by IgE)	COPSAC Results (Atopy by SPT)
2	rs1367180	Dietary	6 years	Atopy	T	VDAART: 0.009 COPSAC: 0.004	0.63 (0.46, 0.84) *p* = 0.002	0.77 (0.56, 1.05) *p* = 0.10	0.73 (0.46, 1.19) *p* = 0.22	1.20 (0.91, 1.60) *p* = 0.21	1.26 (0.80, 2.00) *p* = 0.32	2.56 (1.19, 5.50) *p* = 0.02
2	rs958457	Plasma	18 months in COPSAC/3 years in VDAART	Atopy	G	VDAART: 0.007 COPSAC: 0.01	2.21 (0.76, 7.07) *p* = 0.16	1.24 (0.90, 1.71) *p* = 0.19	0.99 (0.59, 1.66) *p* = 0.96	0.10 (0.01, 0.60) *p* = 0.02	0.74 (0.49, 1.10) *p* = 0.14	0.39 (0.17, 0.89) *p* = 0.03
2	rs1516311	Plasma		Atopy	A	VDAART: 0.003 COPSAC: 0.02	1.59 (0.65, 4.04) *p* = 0.32	1.05 (0.80, 1.36) *p* = 0.74	0.90 (0.56, 1.42) *p* = 0.64	0.02 (0.001, 0.31) *p* = 0.01	0.68 (0.32, 1.41) *p* = 0.30	0.22 (0.05, 0.91) *p* = 0.04

Abbreviations: SPT = skin prick test.

## Data Availability

Genotype data from VDAART are part of the ECHO consortium and ECHO consortium members can obtain the data directly from the ECHO DCC or, for those not part of ECHO, directly from the authors. All other relevant data are available from the authors upon reasonable request.

## Supplementary Materials

The following supporting information can be downloaded at: https://www.mdpi.com/article/10.3390/nu15102416/s1, References [25,26,27,28,29,30,31,32,33,34,35,36,37,38,39] are cited in Supplementary Material.
