# Supplementary material for: Omega-3 Fatty Acids Interact with DPP10 Region Genotype in Association with Childhood Atopy
Source: Nutrients. 2023 May 22;15(10):2416. doi: 10.3390/nu15102416 (PMC10223962; doi:10.3390/nu15102416)
Supplement: Supplementary file 1 [file nutrients-15-02416-s001.zip › nutrients-2272263-supplementary.docx]

**Supplementary Materials**

**Supplementary Methods**

*Study Participants and Setting*

Analyses were performed using data from the VDAART clinical trial and replication of findings was sought in data from the COPSAC clinical trial.

VDAART[14] is a double-blind placebo-controlled study on the effects of prenatal vitamin D supplementation on asthma and allergy in offspring performed in three sites in the United States: Boston University, Massachusetts; Washington University at St. Louis, Missouri; and Kaiser Permanente Southern California Region, San Diego, California. All participating mothers had a history of asthma, eczema, or allergic rhinitis, and/or had a partner (biological father of the infant) with a history of asthma, eczema, or allergic rhinitis. The study protocol was approved by the institutional review boards at each participating institution and at Brigham and Women’s Hospital. All participants provided written informed consent.

COPSAC[6] is a Danish birth cohort study on the development of asthma and allergies. A subset of study subjects participated in clinical trials of omega-3 fatty acid and vitamin D supplementation during pregnancy on asthma and allergy outcomes in offspring. Unlike VDAART, offspring of COPSAC participants are not at elevated genetic risk of atopic disease. The COPSAC2010 study was approved by the local ethics committee with a separate approval for the vitamin D during pregnancy RCT, by the Danish Data Protection Agency, and by the Danish Health and Medicines Authority. Written and oral informed consent were obtained at enrollment of participants.

*n-3 PUFA Measurement*

*Dietary n-3 PUFA (VDAART):* When offspring were 3 and 6 years old, parents were asked to complete a modified version of a semi-quantitative FFQ that was previously validated in preschool-age children[15]. We used FFQ responses to estimate daily calorie and PUFA intake as previously described[16]. Nutrient compositions of FFQ items were determined using the Harvard nutrition composition database, which is based on US Department of Agriculture publications supplemented by other publications and information from manufacturers[25–27]. A summary variable was created for n-3 PUFA intake by taking the sum of EPA and DHA estimated intakes. To account for total energy intake, nutrient density was calculated for each nutrient by dividing nutrient intake by total calorie intake and all analyses of nutrient densities included estimated calorie intake as a covariate[17].

*Plasma n-3 PUFA (VDAART and COPSAC):* Offspring of participants in VDAART and COPSAC provided plasma samples for metabolomics analysis at age 3 and 6 years (VDAART) and 18 months and 6 years (COPSAC). For both cohorts, metabolomic profiling, mass spectrometer platforms, sample extraction and preparation, instrument settings and conditions and data handling were performed at Metabolon, Inc. (Research Triangle Park, NC). A summary variable was created for n-3 plasma PUFA by taking the sum of EPA and DHA. An additional summary variable was created for n-6 plasma PUFA by taking the sum of adrenate, arachidonate, dihomolinoleate, docosadienoate, n-6 docosapentanoate, hexadecadienoate and linoleate. This value was log-transformed and pareto-scaled in VDAART and scaled to mean 0 and variance 1 in COPSAC.

*Clinical Outcomes and Covariates*

*Asthma:* In VDAART, the outcome of asthma at age 6 years was based on parent-reported physician diagnosis of asthma at any time in the first 6 years of life, and either parental report of wheeze or asthma medication use between age 5 and 6 years. In COPSAC, asthma ascertained at age 6 years was diagnosed based on a previously detailed quantitative symptom algorithm requiring all of the following criteria: (1) verified diary recordings of 5 episodes of troublesome lung symptoms within 6 months, each lasting at least 3 consecutive days; (2) symptoms typical of asthma including exercise-induced symptoms, prolonged nocturnal cough, and persistent cough outside of common colds; (3) need for intermittent rescue use of inhaled β2-agonist; and (4) response to a 3-month course of inhaled corticosteroids and relapse upon ending treatment.

*Atopy:* In VDAART, aeroallergen sensitization was defined by a positive serum specific IgE > 0.35 kU/L at age 6 years to one or more of the following: *Dermatophagoides pteronyssinus*, *Dermatophagoides farinae*, cat dander, dog dander, grass pollen mix, German Cockroach, and *Alternaria Alternata* and/or total IgE of at least 60 kU/L. In COPSAC, atopy by skin test was based on skin test wheal at least 3mm larger in diameter than negative control to at least one of 10 common inhalant allergens in Denmark; and atopy by IgE was defined by serum specific IgE level ≥ 0.35 IU/ml to common inhalant allergens in Denmark.

*Genotyping and Candidate Gene Selection*

Genotyping was performed in VDAART and COPSAC participants using the Illumina Infinium HumanOmniExpressExome Bead chip. Imputation for VDAART was performed using Minimac[28] on the Michigan Imputation Server with the 1000 Genomes reference panel[29]. Genotype principal components analysis (PCA) was performed in VDAART using LASER, which analyzes sequence reads of each sample and place the sample into a reference PCA space constructed using genotypes of a set of reference individuals[30]. In COPSAC PCA was performed using PLINK genotype data imputed on the reference panel of Haplotype Reference Consortium. We selected the candidate genes *FADS, ELOVL2, ELOVL5, DPP10, PTGES,* and *PTGS2* based on evidence of associations with both PUFA and with asthma and allergy with details available in the **Supplementary Materials**.

*Statistical Analysis*

Analyses were performed using PLINK version 1.9 and R statistical software version 4.0.3. Model covariates were selected *a priori*. Covariates in analyses of VDAART data were sex, race (black vs non-black), study center, VDAART treatment assignment and the top four genotype PCs. Covariates in COPSAC data were sex, COPSAC treatment assignment and the top four genotype PCs. Race was not included as a covariate in COPSAC analyses because the vast majority of subjects were white and only children of European ancestry were included in the analyses. Analyses of dietary n-3 PUFA were additionally adjusted for estimated daily calorie intake. To test the hypothesis that genetic variants modify the association of n-3 PUFA with asthma and/or atopy, we performed logistic regression analyses under an additive genetic model including main and interaction effects of genotype and n-3 PUFA.

These analyses were performed on SNPs within 50,000 base pairs of candidate genes in targeted analysis, and on a genome-wide basis. Findings were considered statistically significant on a genome-wide basis if they reached a level suggestive of statistical significance (*p*<1e-5) in VDAART and reached a level of statistical significance with *p*<0.01 and the same direction of association in COPSAC. Candidate gene analysis results were considered statistically significant if they yielded *p*<0.01 in VDAART and *p*<0.05 in COPSAC with the same direction of association. LD clumping was performed using results from VDAART to carry forward only independent SNPs for replication attempts using the function ld_clump in R package ieugwasr. Independent SNPs were those selected by ld_clump using either EUR or AFR population data from the 1000 Genomes Project[29]. For these reported odds ratios, dietary n-3 PUFA nutrient density was divided by 5 for interpretability and comparability with the scale of plasma n-3 measurements.

**Rationale for Candidate Gene Selection**

*FADS* region *FADS1* and *FADS2* encode rate-limiting desaturase enzymes in both n-3 and n-6 PUFA pathways are are located in the *FADS* region on chromosome 11. Multiple studies have found associations between *FADS* SNPs and both PUFA levels and eczema[9–11], and prior studies have also found that *FADS* genotype modifies associations of dietary PUFA intake with hay fever[7], eczema[7], and asthma[6–8].

*ELOVL2* and *ELOVL5* These genes encode elongation enzymes that participate in metabolism of both n-3 and n-6 PUFA. Genetic variants in these genes have been associated with circulating PUFA levels[31], and so could determine tissue-level bioactivity of circulating/ingested PUFA and thereby modify associations of PUFA with clinical outcomes.

*DPP10* The gene *DPP10* has been associated with asthma in prior studies[21–24]. It encodes dipeptidyl peptidase like 10, a membrane protein that binds specific voltage-gated potassium channels and modulates potassium trafficking, and has been thought to possibly impact neurologic control of smooth muscle function in asthma[19]. *DPP10* has not been, to our knowledge, previously linked directly to PUFA metabolism; however, a gene by n-3 interaction study of pulmonary function in adults found an interaction on forced vital capacity (FVC) between the n-3 docosahexaenoic acid (DHA) and a SNP in DPP10[19].

*PTGES* This gene encodes prostaglandin E synthase, which is the terminal enzyme in COX-2-mediated production of prostaglandin E2 from the upstream n-6 metabolite arachidonic acid. Deficiency of pulmonary prostaglandin E2 predisposes to airway hyperresponsiveness and allergic inflammation in humans and in mouse models[32,33], *PTGES* genotype has been associated in GWAS with asthma[34]. Compellingly, genetic variants in *PTGES* interact with fish intake on the outcome on colorectal adenoma[35].

*PTGS2 PTGS2* encodes cyclooxygenase and catalyzes production of inflammatory prostaglandins, which have well-known roles in allergy and asthma pathobiology[36]. Interactions have been observed between dietary fatty acid intake and *PTGS2* SNPs on the outcomes of rectal cancer[37] and prostate cancer[38,39].


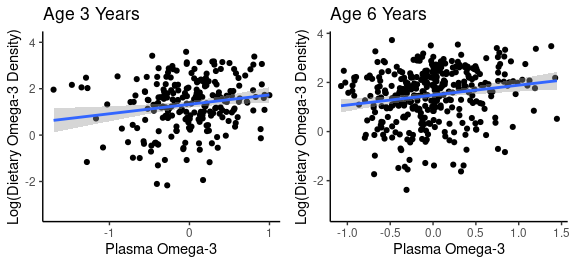


**Figure S1.** Scatterplots of association between plasma n-3 and dietary n-3 density in VDAART participants.


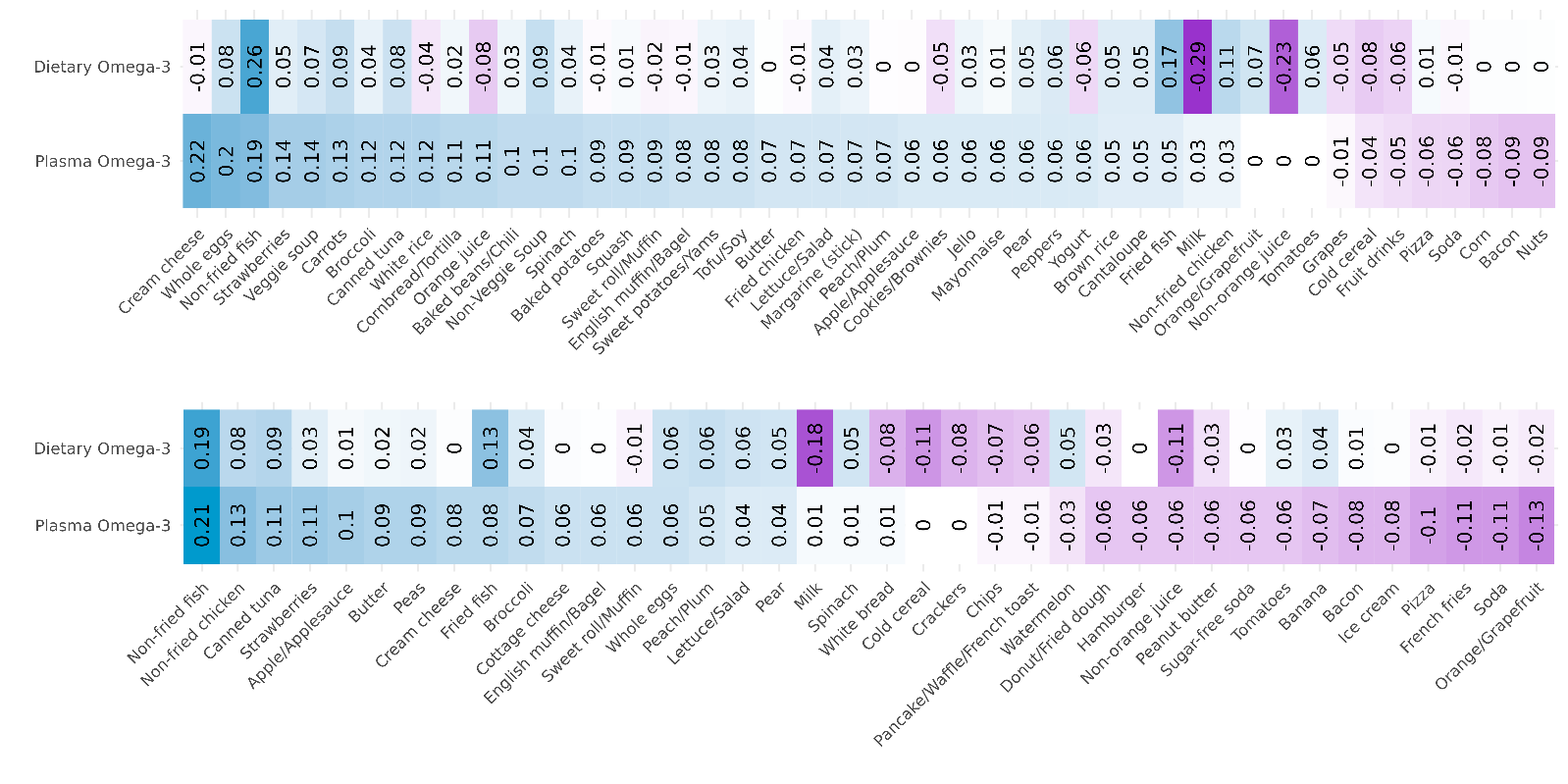
**Figure S2. Heatmaps of food frequency questionnaire item associations with plasma and dietary n-3 in VDAART participants at ages 3 years (Upper Panel) and 6 years (Lower Panel).** Spearman rho values are displayed for correlations of plasma n-3 with food items and only foods with rho > 0.05 for either plasma or dietary n-3 are shown. Beta coefficients from linear regression analyses adjusted for total calorie intake are displayed for associations of dietary n-3 with food items.

**
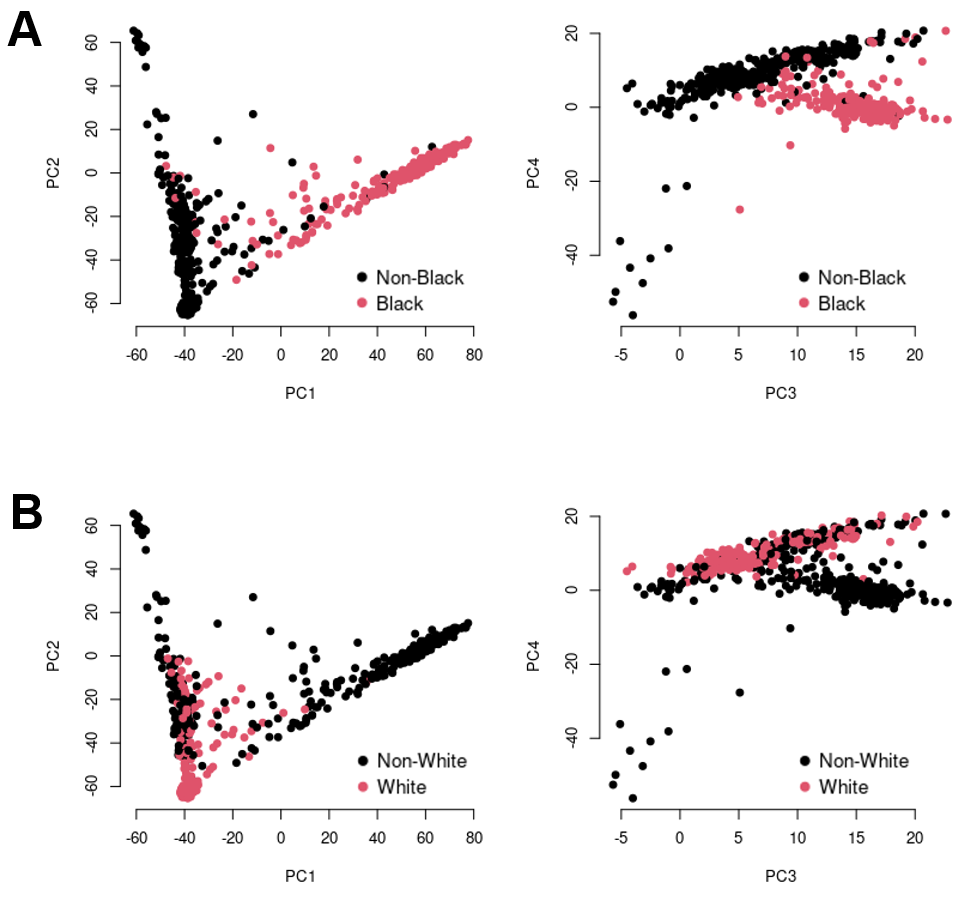
**

**Figure S3.** Top genotype principal components by black vs non-black race (panel A) and by white vs non-white race (panel B) in VDAART participants.

**
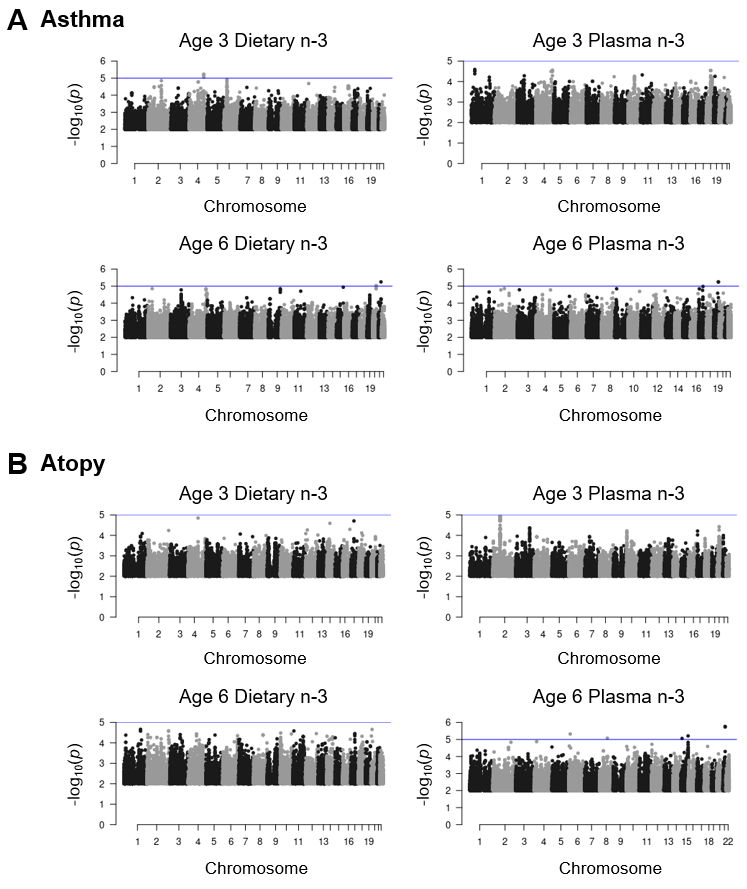
**

**Figure S4.** Manhattan plots of dietary and plasma n-3 interactions with genotype on the outcomes of asthma (panel A) and atopy (panel B).
